# Measuring Environmental Justice in Real Time: A Pilot Study Using Digital Participatory Method in the Global South, Nepal

**DOI:** 10.3390/ijerph19084752

**Published:** 2022-04-14

**Authors:** Rehana Shrestha, Klaus Telkmann, Benjamin Schüz, Pramesh Koju, Reshma Shrestha, Biraj Karmacharya, Gabriele Bolte

**Affiliations:** 1 Leibniz Science Campus Digital Public Health Bremen, 28359 Bremen, Germany; benjamin.schuez@uni-bremen.de (B.S.); gabriele.bolte@uni-bremen.de (G.B.); 2 Institute of Public Health and Nursing Research, University of Bremen, 28359 Bremen, Germany; telkmann@uni-bremen.de; 3 Department of Public Health and Community Programs, Kathmandu University, Dhulikhel 45200, Nepal; kojupramesh@kusms.edu.np (P.K.); birajmk@kusms.edu.np (B.K.); 4 Department of Geomatics, Kathmandu University, Dhulikhel 45200, Nepal; reshma@ku.edu.np

**Keywords:** environmental inequalities, environmental exposure, perceived fairness, ecological momentary assessment, smartphone

## Abstract

Individuals’ perceived fairness or justice beliefs are related to health in numerous ways. However, environment justice research to date has given little attention to perceived fairness of environmental exposures as experienced by individuals. This study explored the feasibility of a bottom-up digital participatory (via mobile phones) approach using ecological momentary assessment (EMA) to capture individuals’ subjective experience of environmental exposures and the subjective evaluation of fairness by those affected in the context of Nepal. In total, 22 individuals participated in the study for 28 days. The results show high rates of study retention and adherence. Individuals’ justice perception was found to vary within and between individuals, but also substantially depending on the types of environmental exposures. Nevertheless, the study indicates that uncertainties are inevitable as study design and timing may conflict participants’ daily lives and priorities. The method allows us to consider multiple geographic contexts of individuals’ everyday lives beyond residential environment. This pilot study proved the possibility to assess perceptions of environmental justice issues and demonstrated the necessary steps to using digital participatory method for assessing subjective perception of fairness of individuals.

## 1. Introduction

The WHO estimates that 24% of the global disease burden and 23% of all deaths are attributable to environmental factors [1]. Environmental risk factors include exposures to pollution and chemicals (air, water, soil, products), physical exposures (noise, radiation), the built environment (buildings, land use, infrastructure) that result in adverse effects on public health [2]. Moreover, large differences in the environmental contribution to various disease conditions exist among countries of the Global North and the Global South. For example, in low- and middle-income countries, 25% of all deaths are attributed to environmental factors, whereas only 17% of deaths are attributed to such causes in high-income regions [1]. In general, the North–South divide is based on its political and socioeconomic dimension. Following this consideration, definitions of the Global North include North America, Western Europe and developed parts of East Asia, while the Global South is made up of Africa, Latin America, developing Asia, and the Middle East [3].

Viewed from an environmental justice perspective, it is now commonly understood that much of the burden of environmental ill health falls disproportionately on socioeconomically disadvantaged people [4]. Moreover, there is now substantial evidence that people from low–middle-income nations are often exposed to higher levels of environmental stress [5], suggesting that it is even more relevant to assess social inequalities in environmental exposures in the Global South. Rapid, haphazard urbanization and motorization happening in the Global South cities together with a lack of environmental management is increasing the gap among the social groups with regard to environmental exposures and thus contributing to global health inequalities. However, scholarly discourse in social inequalities in environmental exposure has generally focused on cities in the Global North, and there is relatively less research assessing social inequalities in environmental exposure within cities in the Global South context [5,6].

From a methodological point of view, in environmental health research, environmental justice is generally analyzed by assessing social inequalities in exposure to environmental burdens and resources. Using a normative concept of justice—social distribution of environmental burdens and resource judged as avoidable and unfair—and by analyzing objective data from a stationary station or mobile sensor or via a questionnaire survey [7,8], researchers report social differences in environmental conditions or exposures. Thus, using top-down perspective researchers define such inequalities as inequity or environmental injustice without explaining the criteria used to assess an observed environmental inequality as inequity. While normative theory provides empirical researchers with a well-stocked toolbox for evaluating visible inequalities and has driven most of the research in environmental justice field to date, it might pose a limitation to fully analyze the possible impact of environmental justice issues on people’s health. One reason might be, as Schuppert and Wallimann-Helmer [9] argue, that in many cases it is not clear “what states of affairs and on the basis of which grounds should the inequalities be labelled injustices, or how different normative concepts such as equality, justice and well-being are connected to each other”. Moreover, accumulating evidence now indicates that individual perceptions of fairness or justice beliefs are related to health in numerous ways such as stress reactivity and coping; these perceived fairness or feelings of justice may affect numerous measures of well-being such as life satisfaction, positive mood, anxiety, depression and general distress [10]. For instance, research indicates that individuals with a high belief in a just world may sleep better [11], have more positive effects, health coping, long-term hope and gratification [12,13,14], and exhibit adaptive functions which promote subjective well-being [15]. Environmental justice research to date has given little attention to a bottom-up perspective considering perceived fairness of environmental exposures as experienced by the individuals and the variation in such perceptions. It has to be clarified whether people who are less aware of their own exposure to negative environmental factors as well as to social differences in living conditions in their society would subjectively assess their situation as being less unfair. This kind of lack of awareness may prevent impairment of their health by reducing stress. Yet, exposures to negative environmental factors will still have a negative impact on physical health outcomes, independent from the subjective awareness.

Here, we use smartphone-based participatory assessment to provide such a bottom-up assessment of environmental justice. Increasing use of smartphones is now providing a new avenue in environmental justice research. The use of mobile apps is enriching the assessment of individuals’ exposures to various environmental factors in their living environment. By embedding sensors in individuals’ mobile phones, researchers are able to measure environmental exposure at the individual level and at higher temporal, spatial and geographical scale [16,17]. In addition to this passive participatory sensing approach where individuals carry sensors that automatically collect data of exposures wherever they go, mobile phones are also creating opportunities for individuals’ active participation—individuals are required to generate the collected information, as prompted by the research application, and decide for themselves to report data such as taking photos, sending a location tag as well as their subjective experience to exposures. For instance, in the Noise Tube approach [18], individuals can use their mobile phone as a noise sensor to measure their own exposure as well as social tag their exposure, meaning they can supply all kinds of qualitative data such as sources of noise, the location, the time and subjective experience such as annoyance.

To the best of our knowledge, however, the existing approaches have not considered explicit recording of the subjective evaluation by those affected regarding the fairness of their exposures. Intrapersonal and interpersonal factors such as an individuals’ ability to cope and sensitivity may further influence how one perceives environment and its subjective evaluation across time and space [8,19]. This suggests the need to account for both within- and between-person variation in the rating of environmental exposures and of judgement of fairness.

To address this gap, the current research aims to examine the feasibility of using the ecological momentary assessment-based [20] digital participatory method in order to capture individuals’ subjective experience of environmental exposures and their subjective evaluation of fairness. The study examines adherence and compliance of participants in the study, assesses perceived environmental exposures and perceived fairness of justice among the participants, assesses between- and within-participants variation across various environmental types and their perceived fairness and eventually presents lessons learned in developing and implementing digitally enabled participatory approach for environmental justice research in the context of the Global South, Nepal. The implication and significance of the result of this study is to inform on various opportunities and practical issues that may need to be considered in a future full-scale study employing the digital participatory method for collecting individuals’ subjective exposures and assessment of fairness. In addition, the study presents the first result of various analyses that could be derived through the collected data.

## 2. Materials and Methods

### 2.1. Study Context

Nepal, which lies in the southeast of Asia, is recognized as one of the fastest-growing countries. Similar to other countries in the global south, Nepal is being confronted with various environmental problems such as air pollution, noise pollution and solid waste issues [21]. Across the nation various environment-related exposures are causing premature deaths and diseases, particularly among the poor and vulnerable groups. This has resulted in an increase in the health costs. It is estimated that an aggregate environmental health related cost is close to 3.5% of the country’s GDP, representing significant burden of Nepal’s economy [22].

Haphazard urbanization and motorization, use of solid biomass fuel for cooking, and lack of proper waste management systems are some of the factors increasing the burden of disease on population of both urban and rural areas in Nepal. Several research studies on the environment have been conducted in recent decades, especially in the Kathmandu valley [23,24]. Environmental pollution such as air pollution and noise pollution is well documented in Kathmandu valley [25,26]. In some areas in Kathmandu valley, particulate matter is found to be ten times higher than the WHO standard [27]. Likewise, studies have found similar evidence around the Kathmandu valley in other municipalities such as Dhulikhel, Banepa, and Panauti, which are 26 km from the Kathmandu and belong to Banepa Valley [28].

### 2.2. Digital Participatory Method Using Ecological Momentary Assessment

The Ecological Momentary Assessment (EMA) approach was adopted in the development of a digitally enabled participatory method. EMA is one of the most widely used methods to collect participants’ real-time experiences as they unfold during daily life [20]. In contrast to other methods such as subjective perception survey where a participant reports at a single point in time or longitudinal panel study, EMA typically results in many observations per participant and over a predefined period of time, usually multiple days to weeks [20]. As this method enables participants to assess the actual moment of interest in their natural and real-life environments and at multiple time points, it has proven advantageous to assess variation both within and between individuals in, e.g., affective state [29], quality of life [30], mobility behavior, or social networks [31]. This method is also being used in environmental health research to assess environmental conditions not only at the participants’ homes, but also routine places visited throughout daily life [32,33,34]. In addition to subjective experiences, EMA can be combined with standard mobile phone sensors such as GPS location services in order to record the exact location an assessment was conducted in.

EMA can assess data via event-contingent (participants initiate a response when some predefined event occurs), signal-contingent or random (participants are prompted at random times to obtain a representative sample of their experiences), and time-contingent scheduling (participants are prompted on a fixed time schedule) [35,36]. We used event-contingent and time-contingent data collection protocols for this study. Participants were instructed to initiate an event-contingent entry using the smartphone-based EMA app whenever they feel exposed to environmental exposure. Time-contingent assessment was used in the study to prompt the participant to answer a control question in order to assess adherence to the study.

### 2.3. Smartphone-Based EMA App and Questionnaires

We used MovisensXS [37] to implement the study design. We programmed an EMA questionnaire which was then distributed to the MovisensXS app on participants’ smartphones after consenting to participate.

The event-contingent questionnaire included 22 questions addressing type and sources of environmental exposures, subjective concerns and subjective perceptions towards fairness of exposures, momentary mood, life satisfaction and perceived health status. Detailed questionnaire is provided in the Supplementary File (S1A). Environmental exposure questions cover five categories of exposures in line with environmental burdens and benefits—noise pollution, odor, air pollution, other environmental pollutants, built environmental factors. Each of these categories included other sub-categories assessing various known sources of exposures. For example, noise pollution exposures consisted of sources from road traffic, road dust, construction works, etc. Additionally, free text inputs were also provided as another subcategory to allow individuals to report other sources of pollution category specific to certain areas.

Subjective concerns were assessed as the perceived effects of own exposures on a 5-point scale (1 = not at all to 5 = very strong) and as perceived effects as compared to others on a 3-point scale (1 = less affected than other, 2 = same extent as others, 3 = more affected than others). Subjective perceptions towards fairness of exposures were asked as perceived fairness of own exposures in comparison to others and fairness in distribution of exposures in their municipality, both asked on a 5-point Likert scale (1 = very fair to 5 = very unfair). The questionnaires for subjective concerns and subjective perceptions were adopted from the study by Bruderer Enzler et al. [38]. Additionally, perceived controllability– individuals’ confidence that they can perform a task to address a given situation— was asked dichotomously (Yes/No). Perceived controllability represents a psychosocial construct that describes generalized beliefs about one’s ability to affect desired outcomes and avoid undesired outcomes [39]. Momentary mood, current state of health was asked on a 5-point Likert scale (1 = very good to 5 = very poor) and life satisfaction on a 5-point Likert scale (1 = very satisfied to 5 = not at all satisfied). Furthermore, individuals were given the possibility to report their exposure as real-time exposure or as past exposure in order to consider that individuals may not be able to report an exposure while driving, for instance. Similarly, individuals could also decide to upload the GPS location of their perceived exposures as well as submit pictures of such exposures. The time-contingent questionnaire included only one question on whether the individual had encountered any environmental exposure on that day, which was answered dichotomously (Yes/No). The individual received this question daily in evening as an alarm question that can be either answered immediately, postponed for later or dismissed.

Socio-demographic questions were asked in the beginning of the study. These questions included age, gender, education level, place of residence. As for the income, three proxy questions were asked—living in a rental room, shared flat or with family, source of study finance, and ability of household to make ends meet. Based on these questions each individual was then categorized as low, medium, or high, giving higher priority to household situation. Both pre- and post-questionnaires further included questions addressing type and sources of environmental exposures, subjective concerns and subjective perceptions towards fairness of exposures, momentary mood, life satisfaction and health similar to the event-contingent questionnaire Supplementary File (S1B).

### 2.4. Study Population: Piloting, Recruiting and Training

The study design was piloted in Bremen, Germany [40]. Our study applied this to the context of Nepal and adapted the original protocol where needed. For example, participants were asked to use their own smartphones. As a result, only those who own or were able to manage the Android-based smartphone were involved in the study. Similarly, subcategories of sources of pollution were adapted to the context of Nepal. 

Participants for the study were recruited from the Kathmandu University in Nepal between end of March 2021 to June 2021. The university supported with establishing first contact with the participants, during which participants were provided with a brief explanation about the study and collected their email addresses and mobile numbers. The first author then contacted interested participants individually, during which they were provided with more detailed information about the study and the duration of participation. They were also asked explicitly whether they own Android-based smartphones or could manage to obtain them for the study period. Informed consent forms were then sent via email to only those participants who own Android-based smartphones and showed general interest in participating.

A one-to-one online meeting for approximately 1 h was set up with each individual who signed the consent form. During the meeting participants were provided with the link to install the MovisensXS app programmed with the study specific questionnaires in their own smartphone. In order to ensure that the app is properly installed in the participants’ mobile, each participant was allowed to trial with the app. They were also given opportunities to ask questions related to the study and questionnaire design. In the end they were provided with the baseline questionnaire to be filled in immediately after the meeting and send it back to the researcher.

Each individual participated in the study between 28 and 30 days, depending on the starting date. For example, some participants immediately started their study while others began their study period the day after the meeting. The overall data collection period for the study lasted for two months. After the study period, the first author set the second online meeting individually with each participant. They were asked about their experience in participating in the study and provided with the post questionnaire to fill in and send it back to the researcher immediately after the meeting. Participants were reimbursed with an amount of 35 euros for participating in the study.

### 2.5. Ethical Consideration

Ethical approval for the study was received from the University of Bremen (ethics vote No. 12032021) as well as from the Kathmandu University Institutional Review Committee (39/2021).

### 2.6. Data Cleaning and Statistical Analysis

Records that were submitted as trial were excluded for all the participants. Incomplete and missing data for each individual were excluded from the final analysis. Six participants changed their place of residence and took part from the new place of residence. Therefore, all the data collected from the earlier location were discarded for those participants, including the baseline questionnaire.

Descriptive analysis was performed for assessing the adherence of the participants to the study approach. Adherence to the study was assessed across various variables such as number of abandoned prompts, i.e., event-contingent prompts that were initiated but were not completed, time-contingent that were missed prompts [41], mismatched records where individuals submitted their exposures but answered otherwise and vice versa. Following the definition of adherence by Schleicher et al. [42], we also assessed days of interaction and interaction continuity for each individual. Days of interaction captures the number of days of user–app interaction allowing for breaks during which the user has no exposure entries. Interaction continuity herein is considered as entries from the day of registration onwards until first break. Additionally, we also assessed the adherence in terms of submitting locations and images.

The relationships between various aspects—subjective concerns, subjective perceptions towards fairness of exposures, momentary mood, life satisfaction and perceived health status—and exposure were analyzed by fitting linear mixed models. For perceived controllability, a logistic mixed effects model was applied. Since data at the subject level cannot be assumed to be independent, random intercepts were included for each participant. As fixed effects we included exposure into the model. No substantial violation of homoscedasticity and normality of the residuals was determined by visual inspection of the residual plots. The between-participant variation was assessed by the intraclass correlation coefficient (ICC). Corresponding 95% confidence intervals were calculated via bootstrapping. To check for differences between exposure levels likelihood ratio tests of the full model against the model without exposure were employed and corresponding *p*-values are reported. All analyses were performed in R version 4.0.2 (R Core Team 2020) using the lme4 package [43].

We performed Wilcoxon signed rank tests to investigate whether there were noteworthy differences on general subjective perceptions after participating in the study with regard to perceived effects due to various environmental types and sources, perceived effects as compared to others, perceived fairness of exposures compared to others, perceived distribution of the exposures in the municipality as well as subjective experiences related to general life satisfaction and general health status.

## 3. Results

### 3.1. Characteristics of Participants (N = 22)

Out of 23 participants who initially volunteered to participate, one participant was excluded as the participant left the study midway, thus resulting in a high retention rate (95.6%). Data from this participant were not included in the following analyses. The final sample (Table 1) included students (N = 22; 13 male and 9 female). The average age of the participants was 23.5 years (SD = 2.9). Participants were predominantly Bachelor’s students, most of them living in urban municipalities, and were mostly from medium-income households.

### 3.2. Adherence to the Study Approach

Table 2 presents the details of adherence of the participants to the study. In total, participants submitted 1040 event-contingent prompts, out of which 2% started but were discontinued or abandoned. Similarly, there were around 4% of mismatched submits, meaning that participants either reported to being exposed although they were not exposed or vice versa. With a total of 616 time-contingent prompts among the 22 participants for 28 days, 9% of prompts were missed or were not responded. On average, participants reported 1.7 exposures per day. High adherence was observed to submitting locations of their exposures (94.9%) as compared to submitting images of such exposures (74.8%). 

As Table 3 shows, there was considerable variability among participants across various aspects of adherence. While participants felt exposed on an average of 20 days of participation, they did not report being exposed every day except for one participant who felt exposed every day. Moreover, one participant indicated not being aware of environmental exposures most of the time and, therefore, reported exposures only twice over 28 days. On average, participants continued exposure entries for 6 days from the day of registration onwards until the first break, except for one participant who did not have a break throughout the study duration. Variability in number of self-reports was also high, with number of reported exposures ranging from 2 to 174, but with only one participant reporting 2 and 174. Participants were less adherent to submitting an image than submitting the location of their exposures. 

### 3.3. Perceived Environmental Exposures by Type and Sources

**Figure 1:**
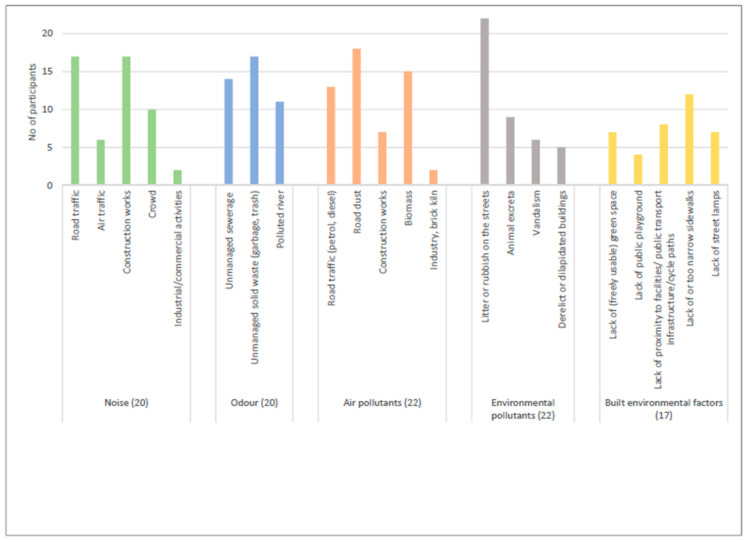
Number of participants who reported various types and sources of exposures.

Figure 1 shows the perceived exposures to various environmental factors and sources in the daily lives of the participants. 

**Figure 2:**
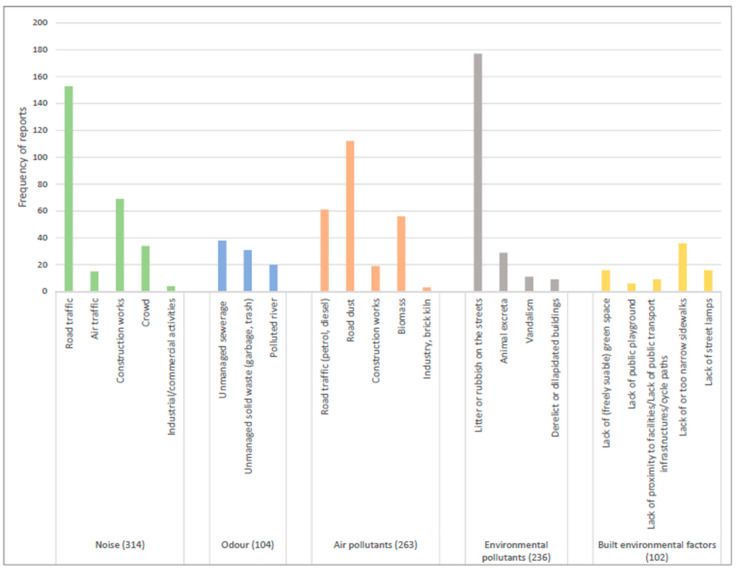
Frequency of exposures reported across various types and sources of exposures. Note: In Figure 1 and Figure 2, only those sub-categories for which there are reported exposures are presented and some of those subcategories within the same categories are grouped together due to few reported exposures.

All participants perceived exposures to air pollution and environmental pollutants in their respective municipality whereas two participants did not perceive exposure to noise and odor. With respect to the sources in each of the environmental categories, all participants reported litter or rubbish on the streets as one of the major sources of environmental pollution in their municipality (Figure 1). This source of environmental pollution was also reported frequently over 28 days (Figure 2). More than three quarters of the participants were exposed to road dust as a major source of air pollution (N = 18) followed by road traffic noise (N = 17) and construction noise (N = 17) as sources of noise pollution. However, relatively higher frequency of exposures was reported for noise pollution especially caused by road traffic rather than for air pollution (Figure 2).

### 3.4. Subjective Concerns and Subjective Perceptions towards Fairness of Exposures across Environmental Types

**Figure 3:**
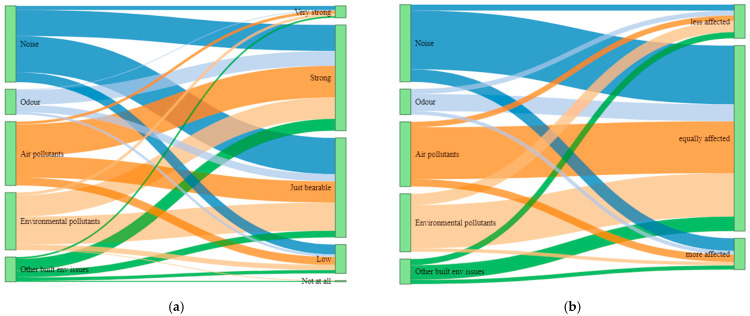
Distribution of reported exposures on (**a**) to what degree they are perceived to have effects on oneself; (**b**) to what degree they are perceived to have effects on oneself as compared to others.

Most of the reported noise pollution and environmental pollutants were perceived as just bearable, as shown in Figure 3a, by more than half of the participants (Table S1), whereas strong effects were perceived towards most of the air-pollution- and odor-related exposures by more than half of the participants (Table S2). When compared to others, these exposures are mostly perceived to be affecting oneself equally as others (Figure 3b). These are reported by the majority of participants, particularly for noise, air, odor and environmental pollutants (Table S2). Yet, in a few instances of exposures, almost half of the participants perceived that their exposure was affecting oneself more than others except for built environment-related factors (Table S2).

**Figure 4:**
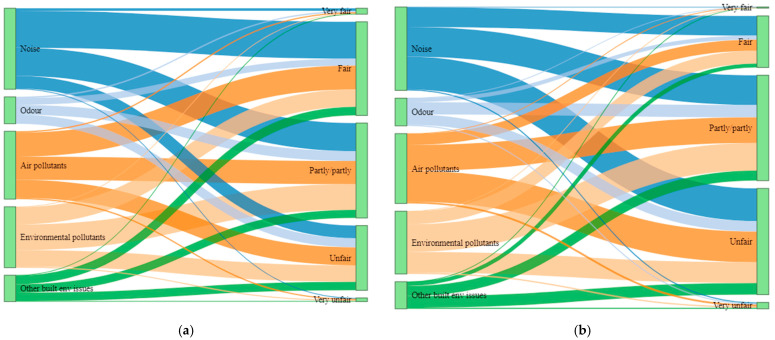
Distribution of reported exposures on (**a**) to what degree they are perceived to be fair as compared to others; (**b**) to what degree they are perceived to be fairly distributed in respective municipality.

In most of the exposure instances and across all types of environmental factors, participants perceived their exposures to be either fair or they perceived them neutrally (Figure 4a) and these responses were indicated by more than half of the participants (Table S3). On the contrary, most participants did not perceive the distribution of those exposures to be fair. In an almost equal number of instances of exposures and across all environmental factors, they mostly perceived them to be either unfairly distributed or rated them neutrally (Figure 4b) and these responses were indicated by half of the participants except for built environmental factors (Table S4).

Intraclass correlation coefficients along with their 95% confidence intervals for perceived effects and perceived fairness of exposures are presented in Table 4. In particular, these coefficients serve as an estimate for the proportion of total variance that is explained by participant clustering. It shows that perceived effects as compared to others do not vary much among individuals but within individuals. Similarly, ICCs indicate moderate to high variation in perceived effects and perceived fairness of exposures among individuals as well as in perceived fairness of distribution of those exposures. These findings also justify the application of a mixed effects model.

**Figure 5:**
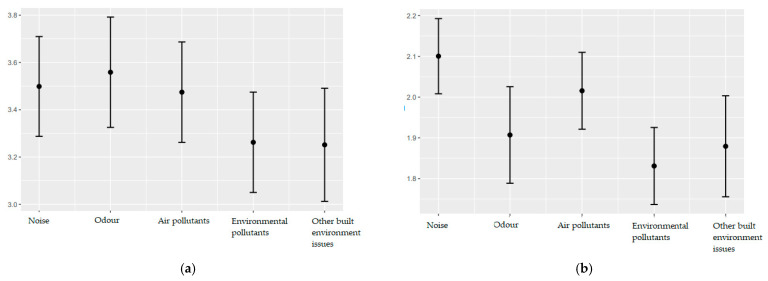

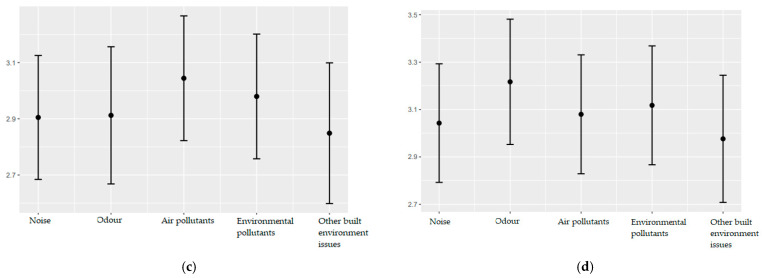
Estimates and 95% confidence intervals across five environmental exposures for (**a**) perceived effects due to exposures, (**b**) perceived effects as compared to others, (**c**) perceived fairness of exposures as compared to others, and (**d**) perceived fairness of distribution of exposures in municipality.

Perceived effects and perceived fairness may be situational and depend on the type of exposure. Therefore, five different types of exposures were included as fixed effects in the linear mixed effects models. Estimates and corresponding 95% confidence intervals are presented in Figure 5a–d. Including exposure in the model led to a significant increase in model fit for perceived effects due to exposure as assessed by a likelihood ratio test (χ^2^ (4) = 27.094, *p* < 0.001) compared to the null model. In particular, participants felt slightly stronger effects by noise, odor and air pollutants than by environmental pollutants and other built environmental issues as shown in Figure 5a. Additionally, there were strong differences between different types of exposure when it comes to judging if oneself is more or less affected than others (χ^2^ (4) = 40.389, *p* < 0.001). There is a trend towards feeling more affected than others by noise as depicted in Figure 5b. Individuals’ perceived fairness of exposures was not found to differ systematically across environmental exposure types (χ^2^ (4) = 8.329, *p* = 0.7822) (Figure 5c). With regard to perceived fairness of distribution of exposures in municipality, although a likelihood ratio test suggests that inclusion of exposure type led to a significantly better model fit (χ^2^ (4) = 0.02743, *p* < 0.05), no major differences between these exposures could be found (Figure 5d). In this case, odor was perceived to be distributed slightly more unjust in their respective municipalities.

### 3.5. Subjective Perceptions on Controllability across Various Types of Exposures

**Figure 6:**
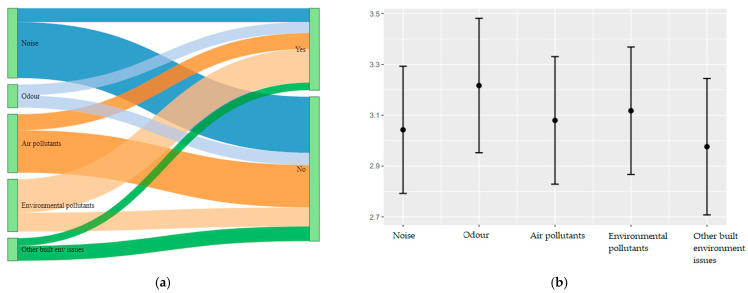
(**a**) Distribution of reported exposures on whether participants perceived to have control on those exposures; (**b**) estimated probabilities of participants and 95% CIs on reporting positive controllability across various environmental types.

In this line, as shown in Figure 6a participants reported to have no control on the majority of instances of noise exposure, air pollution and built environmental factors and these are reported by more than half of the participants (Table S5). However, a majority of participants perceived that they had control on exposures due to environmental pollutants. Moreover, there were systematic differences in perceived controllability of different types of exposures (χ^2^ (4) = 120.06, *p* < 0.001) where environmental pollutants were perceived as most controllable and noise the least as shown in Figure 6b.

### 3.6. Momentary Mood, Life Satisfaction and Subjective Health Status

**Figure 7:**
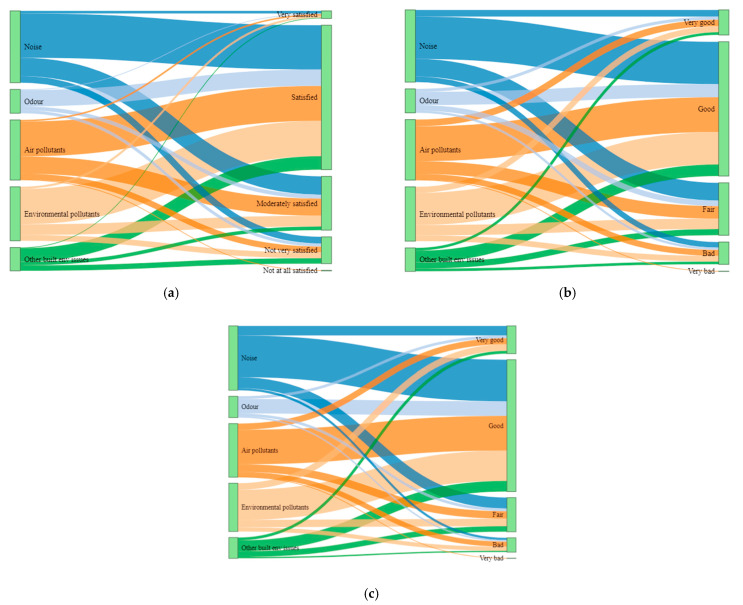
Distribution of reported exposures across (**a**) momentary perceived life satisfaction; (**b**) momentary perceived mood; (**c**) momentary perceived health status.

In general, participants evaluated their momentary experiences related to life satisfaction, mood and health status positively in most of the instances of exposures across all the types of exposures (Figure 7a–c). The ICCs indicate that these experiences, however, vary among individuals substantially (Table 5), but they do not vary significantly across the environmental types as is assessed by likelihood ratio tests (Figure S1a–c).

### 3.7. Subjective Perception before and after the Study (N = 22)

The findings from before and after study comparison of perception of individuals do not show significant changes in the subjective perception of individuals on most of the variables, namely, perceived effects of exposures when compared with other people, perceived fairness of own exposures, perceived fairness of distribution, subjective life satisfaction and health as assessed by paired Wilcoxon signed rank tests (Table 6). However, there are significant changes in perception of effects of exposures related to a number of sources of noise pollution and sources of odor. In general, participants reported higher perceived effects due to exposures in the post-study questionnaire for all sources of environmental exposures across all environmental types except for built environmental factors.

### 3.8. Qualitative Remarks on the Study and Application

Participants were asked about their experience in participating in the study in the post questionnaire, which they shared on two aspects–significance of this study for oneself and for the city administration and the usability aspects of the digital application. Increasing awareness of their environment own exposures were reported frequently as is reflected from participants remarks

“…during and after the participation, I become more conscious about the environmental hazard nearby.” (ID 4)

“..I felt a difference in the way I used to look at the things around.” (ID29)

Moreover, they see the potential of such a method in creating consciousness among individuals for taking actions or informing authorities about the situation.

“citizens can directly report about the different pollutions and built environmental issues …so that municipality can easily solve the problem.” (ID24)

“..I feel motivated to do something about the pollution eventhough it makes a small difference.” (ID7)

With regard to technical aspects of the employed method, most participants found it easy to use and did not feel burdensome. Nonetheless, they suggested on having a possibility to take video with an audio track, especially to record noise exposure. 

## 4. Discussion

We explored the feasibility of a digital participatory method using EMA to record perceived individual exposures to environmental injustice and their subjective perception of fairness of those exposures. Our results suggest that the method is feasible for identifying multiple types and sources of environmental burdens that individuals are exposed to in their daily lives. This adds to current research in adding a subjective and real-time perspective to environmental exposures of individuals [34,44,45]. As environmental justice research has often used normative perspective that would define top-down what injustice is, whereas the method adopted in this research might provide a more bottom-up perspective on experienced and perceived environmental inequalities and injustices.

### 4.1. Key Findings

Our study shows that a dynamic approach to environmental justice that examines the day-to-day variation in subjective perception can be implemented by employing a digital participatory method. More importantly, the study revealed that individuals’ subjective perception with regard to exposures and justice vary not only between individuals but that these perceptions also vary within their daily lives. This highlights the importance of using dynamic methods of assessment and addresses previous claims that environmental pollution is highly variable in space and time resulting into objective exposures to vary between (or across) individuals in a given city (due to different locations, personal activities, residential and lifestyle factors, etc.) and within individuals over time (due to mobility, day-to-day differences in personal activities, etc.) [46,47].

Furthermore, variation between (across) individuals’ perceptions were found to be situational depending on the types of environmental exposures. Although littering was perceived as the most visible sign of environmental pollution and was reported by all the participants frequently over the period of 28 days in the study context, the results on subjective effects and fairness show otherwise. Findings suggest a general trend of noise exposures, odor, air pollution to have stronger effects on individuals, noise related exposures on judging oneself being affected more than others, whereas odor-related exposures were perceived to be unjustly distributed. Based on the literature we may argue that having young adults as in this study could have been an important factor when an individual ascribes stronger effects to some of the exposures considered. For example, studies comparing noise annoyance among socioeconomic groups found strong noise annoyance related to psychosocial distress among young adult rather than in older adults [48]. Moreover, Maris et al. [49] argued that “if the exposed has little control over the source, or little trust in the source, the perceived coping resources will be reduced and psychological stress will arise”. In this line, as indicated by results since individuals ascribed less control over certain environmental exposures than others, this could have potentially led to situational variation in perceptions of effects and perceived fairness. 

### 4.2. Methodological and Practical Considerations

#### 4.2.1. Technology Related Challenges and Needs

The study was run on participants’ own smartphones. This decision was driven by three reasons. Firstly, there is an increasing evidence that use of mobile among general public in the Global South context is becoming commonplace [50,51]. Secondly, previous research has indicated that participants usually prefer to carry one single device and people have difficulty keeping two devices charged [41,52]. Finally, providing individual mobiles for participants in a full-scale study becomes eventually resource intensive, which also counteracts the very benefit that we are trying to achieve by using smartphone apps for participatory assessment in environmental justice research.

Our study showed that interoperability of assessment tool is important, as not everyone used Android smartphones. Nonetheless, most of the participants contacted for the pilot study owned Android smartphones and three out of the four iOS users were able to manage to obtain another device with Android. However, supporting a wider range of smartphone models and operating system versions using off-the-shelf EMA apps, persisted as a technical challenge especially for collecting time-contingent assessment during the study. While one participant did not receive the time-contingent prompt throughout the study duration, others received them intermittently indicating that interaction of EMA app with other installed apps may have led to scheduled time-contingent assessments failing to launch and prompts being missed. Additionally, as indicated in another study [41], non-study-related uses of the personal smartphones also affect the ability of the EMA app to function as intended. By looking at data logs and by talking to the participants, we found that participants often forgot to turn off the vibration mode leading to time-contingent assessments being missed. By identifying this problem at an early stage, we opted to send time-contingent emails to the participants and were able to receive a 90% response rate.

#### 4.2.2. Participants Involvement, Retention, Study Duration and Coping with Uncertainties

This study was piloted by recruiting university students aged between 19–30 years. Strategy to recruit participants via university contact was found to benefit the study particularly to generate interest and willingness among the participants, and to retain even when the study encountered uncertain situation. For example, due to closing of the university in response to corona related lockdown measures (mid-April, 2021 to mid-June, 2021), six participants out of 23 moved back to their home in the middle of the EMA study. Nonetheless, five participants began the study from their new place of living suggesting high degree of willingness and general interest in the study. 

The study showed that young people are good candidates for maintaining adherence to the study protocol, as they are regular smartphone users. However, regardless of their technology experiences, having a one-to-one session with each individual proved to be essential and useful to train them in using smartphones for completing EMA prompts. By allowing them to trial with one event-contingent submit during the session, the researcher was able to support the participant in resolving a few of the problems encountered while setting up their personal smartphone. Through the pilot study we also learned that instant access and monitoring to information regarding participants’ completion of EMA survey was necessary and resonate with others [41]. With few of the participants, there appeared to be problems, such as no exposure being recorded for a number of days. The researcher contacted the participants to determine the problem, which was mostly related to failure to automatically upload EMA records to the server; this problem was resolved by reminding the participant about the manual upload option in the app. Moreover, a one-to-one session via online meeting has proven to be successful with young participants.

Unlike a perception-based survey, where participants report at one point in time, research enabling active participation through smartphone and demanding repeated assessment for a longer duration may become burdensome, affecting the adherence to the study. Therefore, high rates of study retention can be expected to relate to duration as is often discussed by prior research [53]. For example, EMA studies conducted for a short period such as 5 days or 7 days have reported retention rates of more than 90% [54,55]. Nonetheless, there are also studies reporting more than 70% retention rates in their 6-month and 12-week study period, respectively [41,56]. In this study, we were able to obtain high rates of study retention in a 1-month period, confirming that high rates of retention can be achieved in longer studies. Anecdotally, many participants did not find the 1-month study duration too intensive. They reported on having enjoyed participating in the study as it made them aware of many environmental exposures that were previously ignored. However, our experience suggests that uncertainties are inevitable for such a longitudinal study, as study design and timing may conflict participants’ daily lives and priorities. In addition to few participants restarting the study from a new location, one participant had to take a week off due to personal reasons before resuming again, which was marked and excluded from the data. This indicates the need for the study design to be flexible enough to accommodate for such uncertainties without compromising the quality of the data. Furthermore, flexibility in the design of the study to incorporate participants’ daily lives could potentially lead to motivation and high retention rate. This is particularly important in studies employing a participatory approach to collect self-initiated report of exposure as adherence to responding to the survey is crucial for gaining insights into the real-time exposures of individuals. Additionally, direct monetary incentives could have likely improved the retention rate in this study as is suggested in prior research [57].

#### 4.2.3. Digitally Enabled Participatory Method for Environmental Justice

In this study, we considered the dynamic aspects in the daily lives of individuals and their mobility patterns to capture their real exposures to various environmental factors. Past studies on environmental justice have heavily focused on residence-based assessment, exclusively considering individuals’ home location to measure individuals’ environmental exposure [58,59,60]. Recent research in environmental epidemiology and environmental exposure assessment has recognized that this neglect of human mobility and the spatiotemporal variations of environmental exposures can lead to erroneous results due to the so called uncertain geographic context problem (UGCoP) [34,61,62]. The UGCoP arises when spatial configuration of the contextual areas influencing human health are misrepresented or when timing and duration in which individuals are exposed to environmental risks are ignored [63,64]. Using an EMA-based digital participatory approach allowed us to address the UGCoP in environmental justice research by incorporating human mobility and spatiotemporal variation in the design. It allows us to record environmental exposures and justice perception not only where individuals live in the municipality but also where and when they visit, study, work, thereby including the multiple geographic context of individuals’ everyday lives in the assessment of environmental justice. 

Consideration of individuals’ mobility within an approach means that the individuals can report their exposures at places where they work, play, visit and not necessarily have to be within their place of residence. Having this flexibility has, however, led to some challenges during the study. We realized that individuals’ daily activity patterns may not only traverse areas beyond the residential neighborhood, but they may often do not coincide with the municipality’s jurisdiction. However, owing to the study design to capture how individuals perceive the distribution of those exposures in their respective municipality, we asked participants to record their exposures within their respective municipality. Eventually, fully incorporating individuals’ mobility in our digitally enabled participatory approach was not possible. In this respect, we would argue that the concept of administrative boundary may not be totally discarded as the municipal planning operates within a fixed jurisdiction. 

### 4.3. Strengths and Limitations

This study has some limitations. Firstly, the decision to recruit participants via university contact has limited the participants to adults between 19–30 years old, thereby excluding people of other ages. In this regard, the study population is not representative to the population in Nepal with varying age categories, educational levels and socio-economic backgrounds. Nonetheless, the student population itself is interesting as being a student is commonly experienced as highly stressful and chronic stress due to environmental exposures is likely to increase the risk of underlying disorder [65]. Secondly, in the absence of objective data on individuals’ exposures, this study could not contrast the objective and subjective individuals’ perceptions of exposures. Research has shown that subjective perception of environmental exposures such as air quality and noise are not necessarily consistent with the objective indicators [66,67]. However, with the recognition that policies and programs targeting improvements in emissions or noise exposures may not automatically lead to improvements in the perception of air quality, subjective perception should also be duly acknowledged and combined with objective exposures for informing policies and programs [68]. Furthermore, annoyance due to the perceptions of environmental stressors can impair overall well-being. In the absence of disaggregated objective exposures data common for most of cities in the Global South, we see the opportunities for combining low-cost sensors [17,69] in the current digital participatory method as a solution for collecting concurrent objective data and subjective perception of exposure and justice in place and time. Thirdly, owing to the corona related measures, movements of participants were restricted to varying extent during the study period, which may have influenced the adherence results.

## 5. Conclusions

We explored the feasibility of employing a digitally enabled participatory method using EMA to record perceived exposures of individuals and their subjective perception of fairness of those exposures. We obtained high rates of study retention and adherence response, nonetheless adherence response was found to vary among individuals. Our results suggest that the digitally enabled participatory method is feasible for identifying multiple types and sources of environmental burdens that individuals are exposed to in their daily lives. We found that individuals’ justice perception varies within and between individuals, but also substantially depending on the types of environmental exposures. Several lessons were learned with respect to methodological and practical aspects. Enabling participants to use their own smartphone, which was indicated to be essential, was found feasible. Nonetheless, it is necessary to recognize early on the challenges pertaining to use of a variety of mobile phone models as well as non-study-related use of personal smartphones, thereby devise ways to circumvent such challenges. Our experience suggests that uncertainties are inevitable for such a longitudinal study, as study design and timing may conflict with participants’ daily lives and priorities, indicating the study design needs to be flexible enough to accommodate such uncertainties. Regarding the implication of the method of environmental justice study, our learning suggests that the method allows us to consider multiple geographic contexts of individuals’ everyday lives beyond residential environment. However, this means that the individuals’ daily life is not limited within an administrative boundary of the municipality, which may contradict the municipal planning operating within a fixed jurisdiction. With increasing evidence of use of smartphones among the younger generation than in the older generation [70], the feasibility of using the digital participatory method to perceived individual exposures to environmental injustice and their subjective perception of fairness of those exposures among older people needs to be explored. Nonetheless, this study informed us of the necessary steps to using digital participatory method for assessing subjective perception of fairness of individuals.

## Figures and Tables

**Table 1:** Characteristics of participants.

Characteristics	Sub-Categories	*n*	%
Gender	Male	13	59
	Female	9	41
Education	Bachelor’s education (ongoing)	17	77
	Master’s education (ongoing)	5	23
Academic discipline	Health	10	45
	Engineering	12	55
Current place of living	Urban Municipality	21	95
	Rural Municipality	1	5
Household income	Low	4	18
	Medium	13	59
	High	5	23

**Table 2:** Adherence to EMA prompts (N = 22) by event contingent and fixed time.

Prompt Type	Abandoned ^a^ (*n* = 1040)	Missed ^b^ (*n* = 616)	Mismatched ^c^	Location Submitted (Only for C = Current Exposure)	Images Submitted (Only for C = Current Exposure)	Total
Event contingent (self-assessments)	21 (2.0%)	N/A	39 (3.8%)	793 (94.9%)	625 (74.8%)	1040 (including abandoned),
						1019 (excluding abandoned),
						(C = 836, P = 183)
Time-contingent (control question)	N/A	53 (9.0%)	N/A	N/A	N/A	616

^a^ Initiated the entry but not completed; ^b^ Missed answering time contingent question; ^c^ Mismatch between EMA submits and fixed time; C = current exposure; P = past exposure; N/A = Not applicable.

**Table 3:** Variability of adherence across aspects of adherence.

Prompt Type	Subcategory	Mean	Median	SD	Range
Event contingent (self-assessment)	Days of interaction ^a^	19.8	22	6.49	2–28
	Interaction continuity ^b^	5.7	5	5.7	1–28
	Self-assessment	46.3	41.5	35.12	2–174
	Abandoned ^c^	2%	0%	3%	0–9%
	Mismatched ^d^	1.8	1.0	2.2	0–9
	Location submits	93%	99%	13%	46–100%
	Image submits	74%	80%	29%	0–100%
Time contingent (control question)	Missed ^e^	2.4	1.5	2.8	0–11

^a^ No of days with at least one record; ^b^ No of adjacent days with EMA entries from the day of first entry until first break; ^c^ Initiated the entry but not completed; ^d^ Mismatch between EMA submits and fixed time; ^e^ Missed answering time contingent question.

**Table 4:** Intraclass correlation coefficients on subjective concerns and subjective perception on fairness.

Dependent Variable	Intraclass Correlation Coefficients and 95% Confidence Intervals
Perceived effects due to exposures	0.327 [0.177, 0.462]
Perceived effects as compared to others	0.100 [0.038, 0.173]
Perceived fairness of exposures as compared to others	0.325 [0.175, 0.450]
Perceived fairness of distribution of exposures in respective municipality	0.505 [0.324, 0.637]

**Table 5:** Intraclass correlation coefficients on momentary mood, life satisfaction and health status.

Dependent Variable	Intraclass Correlation Coefficients and 95% Confidence Intervals
Momentary perceived life satisfaction	0.614 [0.430, 0.734]
Momentary perceived mood	0.530 [0.345, 0.667]
Momentary perceived health status	0.663 [0.484, 0.775]

**Table 6:** Before and after change in perceived effects across various types and sources of environmental factors.

Environmental Factors	Diff of Mean	Sig. Test	Environmental Factors	Diff of Mean	Sig. Test
**Noise**			**Environmental pollutants**		
- Road traffic	−0.71	**0.016**	- Litter or rubbish on the streets	−0.23	0.387
- Air traffic	−0.73	**0.003**	- Animal excreta	0	0.903
- Construction works	−0.50	**0.017**	- Urine	−0.27	0.243
- Industry/commercial areas	−0.19	0.430	- Vandalism	−0.48	0.117
**Odour**			- Derelict or dilapidates buildings	−0.32	0.323
- Industry	−0.55	0.062	**Built env. Factors (lack of)**		
- Sewerage system	−0.82	**0.006**	- Green spaces	−0.23	0.396
- Solid waste (garbage, trash)	−0.52	**0.029**	- Proximity to facilities	−0.36	0.131
**Air pollution**			- Playground	0.14	0.536
- Industry	−0.52	0.074	- Public transport infrastructures	−0.27	0.355
- Road traffic	−0.14	0.650	- Cycle paths	0.14	0.496
- Road dust	−0.05	0.941	- Narrow sidewalks	0	0.948
- Brick kiln	−0.50	0.137	- Street lamps	0.27	0.392
- Biomass	−0.36	0.12			

Note: Bold indicates significance at *p* < 0.05.

## Data Availability

Data are contained within the article or supplementary material.

## Supplementary Materials

The following supporting information can be downloaded at: https://www.mdpi.com/article/10.3390/ijerph19084752/s1, S1A: Event-contingent Questionnaires design and logic, S1B: Pre and Post Questionnaire, Figure S1: Estimates and 95% confidence intervals across five environmental exposures for (a) momentary perceived life satisfaction, (b) momentary perceived mood, (c) momentary perceived health status, Table S1: Number of participants indicating to what degree environmental factors are perceived to have effects on oneself, Table S2: Number of participants indicating to what degree environmental factors are perceived to have effects on oneself as comparted to others, Table S3: Number of participants indicating to what degree they are perceived to be fair as compared to others, Table S4: Number of participants indicating to what degree they are perceived to be fairly distributed in respective municipality, Table S5: Number of participants indicating to what degree they perceive their control over exposures across various types.
