# Supplementary material for: Measuring Environmental Justice in Real Time: A Pilot Study Using Digital Participatory Method in the Global South, Nepal
Source: Int J Environ Res Public Health. 2022 Apr 14;19(8):4752. doi: 10.3390/ijerph19084752 (PMC9024717; doi:10.3390/ijerph19084752)
Supplement: Supplementary file 1 [file ijerph-19-04752-s001.zip › ijerph-1618783-supplementary.pdf]

Supplementary File

Measuring Environmental Justice in Real Time: A Pilot Study Using Digital Participatory Method in the Global South, Nepal

Rehana Shrestha [1,2,*], Klaus Telkmann [2], Benjamin Schüz [1,2], Pramesh Koju [3], Reshma Shrestha [4], Biraj Karmacharya [3] and Gabriele Bolte [1,2]

S1A. Event-contingent Questionnaires design and logic.

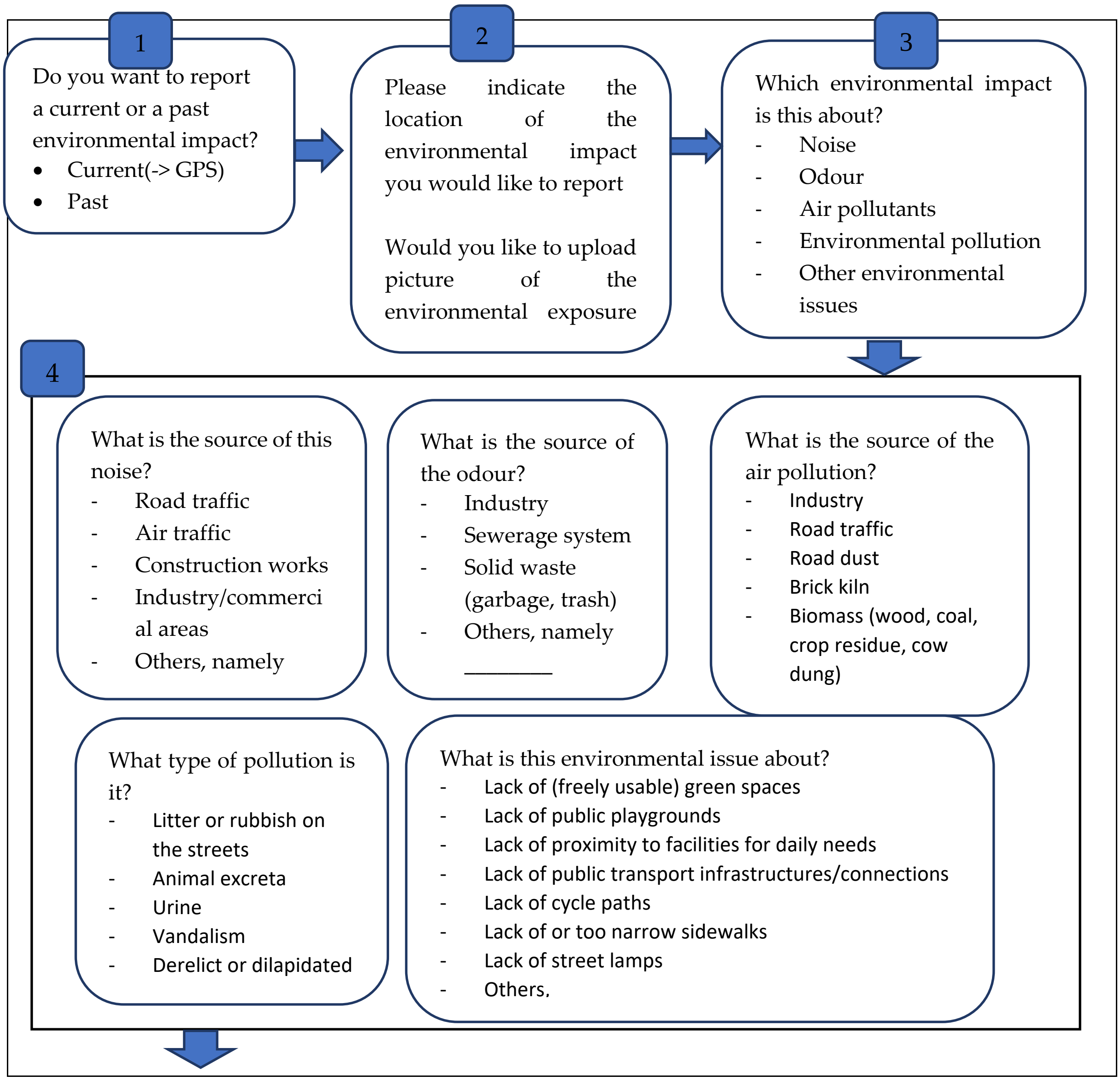

5. How much do you feel affected by this environmental pollution/issues?
- Not at all
- Low
- Just bearable
- Strong
- Very strong

6. Can you do something about this environmental pollution ?
- No
- Yes

7. How do you assess your own concern about this environmental pollution in general when you compare yourself with other people in your municipality?
- I am less affected than others
- I am affected to the same extent as others
- I am more affected than others

8. Do you find your personal exposure to environmental pollution more fair or unfair compared to other people?
- Very fair
- Fair
- Partly/partly
- Unfair
- Very unfair

9. How fairly are environmetnal burdens distributed overall in your municipality?
- Very fair
- Fair
- Partly/partly
- Unfair
- Very unfair

10. How satisfied are you with your life at the moment?
- Very satisfied
- Satisfied
- Moderately satisfied
- Not very satisfied
- Not at all satisfied

11. What is your mood right now?
- Very good
- Good
- Fair
- Bad
- Very Bad

12. How would you describe your current state of health?
- Very good
- Good
- Fair
- Poor
- Very poor

Questionnaires (20-24) were adopted from the follwing study.

Bruderer Enzler, H.; Diekmann, A.; Hartmann, J.; Herold, L.; Kilburger, K.; Kurz, K.; Liebe, U.; Preisendörfer, P. Umweltgerechtigkeit – Soziale Verteilungsmuster, Gerechtigkeits- einschätzungen und Akzeptanzschwellen. Available online: https://data.gesis.org/sharing/#!Detail/10.7802/1993 (accessed on 13.04.2022).

S1B. Pre and Post Questionnaire.

(A) Personal information (only for Pre Questionnaire)

1) What is your gender?

❑ Male

❑ Female

❑ Others

2) What is your birth year?

└┴┴┴┘ (year)

3) What is your highest level of education?

❑ Bachelor education (ongoing)

❑ Bachelor education

❑ Masters education (ongoing)

❑ Masters education

4) What is your academic discipline?

………………………………………………..

5) Where is your current place of living?

❑ Urban Municipality
❑Metropolitan city/municipality ❑Sub metropolitan city/municipality ❑Municipality

❑ Rural Municipality

6) At the moment, whom are you living with?

❑ In a university hostel

❑ In a rental

❑ In a rental room

❑ With friends in a shared flat

❑ With your own family

❑ Others, namely……………………………….

7) How is your study being financed?

❑ Scholarship

❑ Part-time job

❑ Financial support from family

8) A household may have different sources of income and more than one household member may contribute to it. Thinking of your household's total monthly income: is your household able to make ends meet…?

❑ Very easily

❑ Easily

❑ Fairly easily

❑ With some difficulty

❑ With difficulty

❑ With great diffculty

9) How would you describe your state of health in general?

Very good	Good	Medium	Bad	Very Bad
❑	❑	❑	❑	❑

10) Do you have a chronic illness or a long-term health problem? (This means illnesses or health problems that have lasted several years)

❑ No

❑ Yes

11) How satisfied are you with your life in general?

Very satisfied	Satisfied	Moderately satisfied	Little satisfied	Not at all satisfied
❑	❑	❑	❑	❑

12) To what extent the following statement applies to you personally. *Please indicate in each line.*

	**Does not apply at all**	**Apply little**	**Apply somewhat**	**Apply fairly**	**Apply fully**
I can rely on my skills in difficult situations	❑	❑	❑	❑	❑
I can cope well with most problems on my own	❑	❑	❑	❑	❑
I can usually solve difficult task well	❑	❑	❑	❑	❑

13) Do you use a private smartphone?

❑ No

❑ Yes, but not daily

❑ Yes, daily

(B) Environment within your living area

14) How much do you feel affected by noise from the following sources in your current place of living? *Please indicate in each line.*

	**Not at all**	**Low**	**Just bearable**	**Strong**	**Very strong**
Road traffic	❑	❑	❑	❑	❑
Air traffic	❑	❑	❑	❑	❑
Construction works	❑	❑	❑	❑	❑
Industry/commercial areas	❑	❑	❑	❑	❑
Others, namely _ _ _ _ _ _ _	❑	❑	❑	❑	❑

15) How much do you feel affected by odours from the following sources in your current place of living? *Please indicate in each line.*

	Not at all	Low	Just bearable	Strong	Very strong
Industry	❑	❑	❑	❑	❑
Sewerage system	❑	❑	❑	❑	❑
Solid waste (garbage, trash)	❑	❑	❑	❑	❑

Others, namely_ _ _ _ _ _ _ _ _ _ _	❑	❑	❑	❑	❑

**16) How much do you feel affected by air pollution from the following sources in your current place of living?** *Please indicate in each line.*					
	**Not at all**	**Low**	**Just bearable**	**Strong**	**Very strong**
Industry	❑	❑	❑	❑	❑
Road traffic	❑	❑	❑	❑	❑
Road dust	❑	❑	❑	❑	❑
Brick kiln	❑	❑	❑	❑	❑
Biomass (wood, coal, crop residue, cow dung)	❑	❑	❑	❑	❑
Others, namely_ _ _ _ _ _ _ _ _ _ _	❑	❑	❑	❑	❑

**17) How much do you feel affected by any of the following environmental issues in your current place of living?** *Please indicate in each line.*					
	**Not at all**	**Low**	**Just bearable**	**Strong**	**Very strong**
Litter or rubbish on the streets	❑	❑	❑	❑	❑
Animal excreta (dog, cows)	❑	❑	❑	❑	❑
Urine	❑	❑	❑	❑	❑
Vandalism	❑	❑	❑	❑	❑
Derelict or dilapidated buildings	❑	❑	❑	❑	❑
Others, namely _ _ _ _ _ _ _ _ _ _ _	❑	❑	❑	❑	❑

**18) How much do you feel affected by the following issues in your current place of living?** *Please indicate in each line.*					
	**Not at all**	**Low**	**Just bearable**	**Strong**	**Very strong**
Lack of (freely usable) green spaces	❑	❑	❑	❑	❑
Lack of public playgrounds	❑	❑	❑	❑	❑
Lack of proximity to facilities for daily needs	❑	❑	❑	❑	❑
Lack of public transport infrastructures/connections	❑	❑	❑	❑	❑
Lack of cycle paths	❑	❑	❑	❑	❑
Lack of or too narrow sidewalks	❑	❑	❑	❑	❑
Lack of street lamps	❑	❑	❑	❑	❑
Other, namely _ _ _ _ _ _ _	❑	❑	❑	❑	❑

(C) Justice

19) Can you do something about environmental pollution if you feel adversely affected?

- ❑ No
- ❑ Partly/Partly
- ❑ Yes
- ❑ Does not apply, I do not feel disturbed

20) How do you assess your own concern about environmental pollution in general when you compare yourself with other people in your municipality?

- ❑ I am less affected than others.
- ❑ I am affected to the same extent as others.
- ❑ I am more affected than others.

21) Do you find your personal exposure to environmental pollution more fair or unfair compared to other people?

- ❑ Very fair
- ❑ Fair
- ❑ Partly/partly
- ❑ Unfair
- ❑ Very unfair

22) In your opinion, how fairly are environmental burdens distributed overall in your municipality?

- ❑ Very fair
- ❑ Fair
- ❑ Partly/partly
- ❑ Unfair
- ❑ Very unfair

23) Suppose your municipality is going to take measure against environmental pollution. In your opinion, which principle do you think would be most just while implementing measures to protect against the environmental burdens?

❑ Differences in environmental burdens should be balanced as far as possible by the protective measures, so that in the end all citizens have the same burden

❑ All neighbourhoods should be treated equally in terms of protection measures, regardless of current environmental pressures.

❑ The neighbourhood that is currently most polluted should be given priority in protective measures.

❑ The greatest possible number of citizens should benefit from the protection measures, regardless of the current environmental exposures.

24) Have you actively worked to reduce or prevent an environmental impact in the last 5 years?

❑ No

❑ Yes, a signature list or petition signed

❑ Yes, complained to a public authority or to political leaders

❑ Yes, participated in a demonstration or protest action

❑ Yes, participated in a citizens' initiative or local group

❑ Yes, namely_ _

Is there anything else you would like to share with us?

<table>
<tr><td>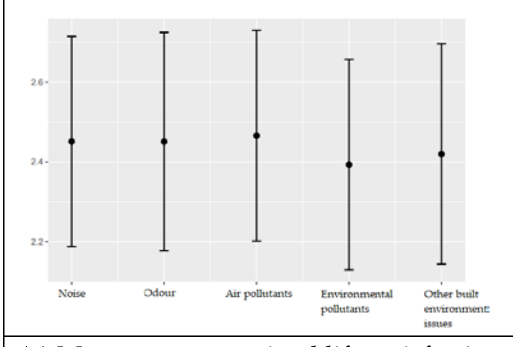
</td><td>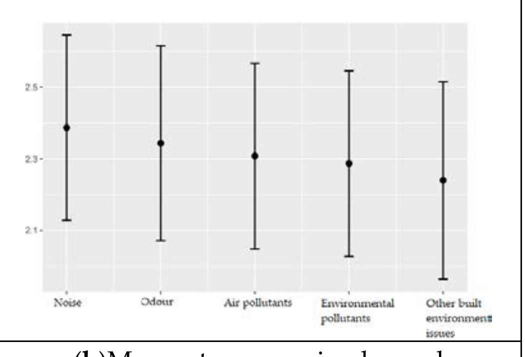
</td></tr>
<tr><td>(a) Momentary perceived life satisfaction
(χ^2(4)= 2.983, p=0.560)</td><td>(b)Momentary perceived mood
(χ^2(4)= 6.527, p=0.163)</td></tr>
<tr><td></td><td></td></tr>
<tr><td></td><td></td></tr>
</table>

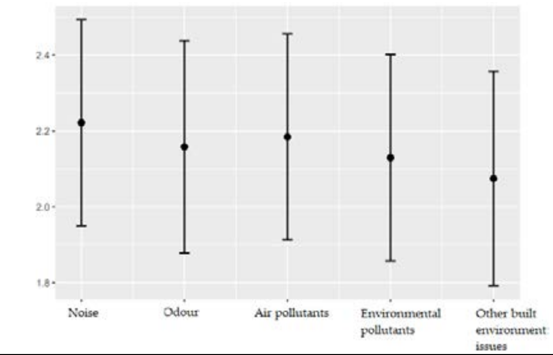

(**c**)Momentary perceived health status
(χ^2(4)= 8.829, p=0.065)

Figure S1. Estimates and 95% confidence intervals across five environmental exposures for (**a**) momentary perceived life satisfaction, (**b**) momentary perceived mood, (**c**) momentary perceived health status.

Table S1. Number of participants indicating to what degree environmental factors are perceived to have effects on oneself.

	Not at all	Low	Just bearable	strong	very strong
Noise	0	9	16	17	7
Odour	0	6	14	15	3
Air Pollution	0	8	17	17	5
Environmental pollutants	1	10	20	17	5
Built Environmental factors	2	8	12	13	2

Table S2. Number of participants indicating to what degree environmental factors are perceived to have effects on oneself as comparted to others.

	less affected than others	affected to the same extent as other	more affected than others
Noise	13	18	15
Odour	11	17	10
Air Pollution	11	21	12
Environmental pollutants	16	21	10
Built Environmental factors	10	14	5

Table S3. Number of participants indicating to what degree environmental factors are perceived to be fair as compared to others.

	very fair	fair	partly/partly	unfair	very unfair
Noise	2	12	20	12	3
Odour	4	13	15	11	0
Air Pollution	2	13	20	12	4
Environmental pollutants	1	13	20	14	3

Built Environmental factors	1	10	12	4	1

Table S4. Number of participants indicating to what degree environmental factors are perceived to be fairly distributed in respective municipality.

	very fair	fair	partly/partly	unfair	very unfair
Noise	1	13	18	12	2
Odour	0	12	12	13	2
Air Pollution	1	13	19	12	2
Environmental pollutants	1	11	17	12	4
Built Environmental factors	1	7	11	7	1

Table S5. Number of participants indicating to what degree they perceive their control over exposures across various types.

	Yes	No
Noise	12	18
Odour	16	14
Air Pollution	15	20
Environmental pollutants	19	16
Built Environmental factors	9	13
